# Investigation of Gelation Techniques for the Fabrication of Cellulose Aerogels

**DOI:** 10.3390/gels9120919

**Published:** 2023-11-21

**Authors:** Natalia Menshutina, Olga Fedotova, Kseniya Trofimova, Pavel Tsygankov

**Affiliations:** Department of Chemical and Pharmaceutical Engineering, Mendeleev University of Chemical Technology of Russia, Miusskaya pl. 9, 125047 Moscow, Russia; chemcom@muctr.ru (N.M.); fedotova.olga.basic@gmail.com (O.F.);

**Keywords:** aerogels, cellulose, gelation, supercritical drying, fibrous structure

## Abstract

Because of the pronounced degradation of the environment, there has been an escalated demand for the fabrication of eco-friendly and highly efficient products derived from renewable sources. Cellulose aerogels have attracted significant interest attributable to their structural characteristics coupled with biodegradability and biocompatibility. The features of the molecular structure of cellulose allow for the use of various methods in the production of gels. For instance, the presence of hydroxyl groups on the cellulose surface allows for chemical crosslinking via etherification reactions. On the other hand, cellulose gel can be procured by modulating the solvent power of the solvent. In this study, we investigate the impact of the gelation methodology on the structural attributes of aerogels. We present methodologies for aerogel synthesis employing three distinct gelation techniques: chemical crosslinking, cryotropic gelation, and CO_2_-induced gelation. The outcomes encompass data derived from helium pycnometry, Fourier-transform infrared spectroscopy, nitrogen porosimetry, and scanning electron microscopy. The resultant specimens exhibited a mesoporous fibrous structure. It was discerned that specimens generated through cryotropic gelation and CO_2_-induced gelation manifested higher porosity (93–95%) and specific surface areas (199–413 m^2^/g) in contrast to those produced via chemical crosslinking (porosity 72–95% and specific surface area 25–133 m^2^/g). Hence, this research underscores the feasibility of producing cellulose-based aerogels with enhanced characteristics, circumventing the necessity of employing toxic cross-linking agents. The process of gel formation through chemical crosslinking enables the creation of gels with enhanced mechanical properties and a more resilient structure. Two alternative methodologies prove particularly advantageous in applications necessitating biocompatibility and high porosity. Notably, CO_2_-induced gelation has not been hitherto addressed in the literature as a means to produce cellulose gels. The distinctive feature of this approach resides in the ability to combine the stages of obtaining an aerogel in one apparatus.

## 1. Introduction

Cellulose, long before its molecular composition was discovered, has been employed by human civilization over millennia in diverse domains, such as construction, textile fabrication, paper manufacture, and as a source of energy. The interest in this biopolymer has not waned, and it continues to be studied and utilized to this day. Advances in nanotechnology have facilitated the development of cellulose-based materials distinguished by their singular attributes, thus broadening the spectrum of potential applications. Noteworthy among these are the fields of biomedicine and pharmaceuticals [1,2], energy storage devices [3,4], flexible electronics [5,6], additive manufacturing [7], biofuel production [8], and sorbent materials [9,10].

Cellulose aerogels exhibit distinct promise across multiple application domains. Aerogels, as a category of highly porous nanostructured materials, are recognized for their low density (ranging from 0.001 to 0.2 g/cm^3^), high porosity (exceeding 90%), and substantial specific surface area (surpassing 200 m^2^/g) [11]. Since their discovery, aerogels based on various materials have been extensively studied. Cellulose aerogels, however, have been developed relatively recently, starting in the early 2000s [12,13], and interest in them continues to grow year by year. The rising interest in these materials can be attributed to escalating demand for environmentally sustainable and renewable resources, the distinctive attributes of their structural composition, and their facile amenability to functionalization.

Cellulose-based aerogels find utility as delivery systems for active pharmaceutical substances (APS). The pronounced porosity and high mesopore volume of the aerogel facilitate substantial loading of APS in an amorphous state, thereby enhancing drug absorption. For instance, in [14,15], a cellulose-based aerogel loaded with resveratrol for osteoarthritis treatment was synthesized. The results demonstrated the efficacy of the APS delivery system in significantly reducing inflammatory factors. In a separate investigation [16], the production of scaffolds utilizing cellulose aerogel loaded with risedronate for bone regeneration was explored. The incorporation of a drug inhibiting bone resorption into porous scaffolds contributes to a more efficacious treatment of bone defects.

In construction, cellulose-based aerogels can serve as structural materials for passive daytime building radiation cooling. In [17], the authors scrutinized the fabrication of such a material. The results indicated that the synthesized aerogels enable the attainment of a maximum cooling temperature of 7.2°C and exhibit an axial compression strength of 1.9 MPa. Over a three-month period, no notable decline in cooling efficiency was observed. The employment of cellulose-based aerogels facilitates a conservation of 52.7% in energy compared with conventional cooling methods.

Another significant realm of application for cellulose-based aerogels is in water purification from pollutants. In [18], a stable, flexible, and hydrophobic sorbent designed for water purification is presented. This sorbent is based on a cellulose aerogel coated with copper nanoparticles. The resultant material exhibits the ability to selectively and expeditiously capture oily pollutants, showing a high sorption capacity and oil absorption rate.

The comprehensive procedure for cellulose aerogel fabrication encompasses the following sequential stages: dissolution of the biopolymer, gelation, solvent exchange, and supercritical drying.

To facilitate the dissolution of cellulose, a solvent is required, one capable of disrupting both the intermolecular and intramolecular hydrogen bonds. The persistence of hydrogen bonds is intrinsic to cellulose’s mechanical properties and is the factor that renders it insoluble in conventional solvents such as water. Cellulose, however, can be effectively dissolved within an aqueous-alkaline system [19]. The hydrated alkali species play a pivotal role in disassembling the hydrogen bonds present in cellulose. In contrast, urea hydrates act to impede the formation of hydrogen bonds within and between cellulose molecules, thereby forestalling the recombination of cellulose macromolecules [20].

The gelation phase exerts a substantial influence on the ultimate material’s structure. Various methodologies can be employed to create cellulose gel. The selection of the gelation approach not only imparts diversity to the ultimate material’s configuration but also influences its structural characteristics.

One of the prevalent techniques for gel formation involves chemical crosslinking. This is realized through an etherification reaction between the hydroxyl groups inherent in the cellulose molecule and a crosslinking agent. Bifunctional compounds, including citric acid [21], epichlorohydrin [22], polyamide-epichlorohydrin [23], divinylsulfone [24], polyethyleneimine [25], and other such agents, are employed for this purpose. The advantages of using chemical crosslinking agents in the production of cellulose aerogels include improving the material’s mechanical properties and increasing structural stability. On the other hand, most chemical substances used as crosslinking agents are toxic, which is a significant drawback. Furthermore, some crosslinking agents may contribute to reducing the material’s porosity and hinder its functionalization potential.

Cryotropic gelation is an alternative technique for gel formation [26]. Cryotropic gelation involves subjecting a polymer-containing solution to cryogenic conditions. This process encompasses the transition from liquid to solid (crystallization) of a low-molecular-weight solvent. Given the disparate freezing temperatures of the solvent and the dissolved substance, two distinct phases emerge. The crystallization of the solvent induces localized supersaturation within the unfrozen phase volume, subsequently leading to the gelation through cellulose coagulation upon immersing the frozen specimens in an anti-solvent, such as ethanol. It is pertinent to emphasize that the freezing stage holds pivotal significance in both the gelation procedure and the ultimate properties of the resultant material. The primary determinants within this process encompass temperature and freezing rate, which dictate the crystallite size within the frozen phase and, consequently, the pore dimensions in the final structure. Thus, this method of gel formation allows for obtaining a highly porous material without the use of toxic substances. However, the disadvantage of this method is the complexity of organizing the process.

A highly promising approach for cellulose gel production is CO_2_-induced gelation [27]. Elevating pressure enhances CO_2_ solubility in water [28], thereby engendering the creation of a weak carbonic acid. This, in turn, leads to a reduction in pH, a phenomenon predominantly responsible for initiating the gelation process. CO_2_-induced gelation has been successfully employed for the generation of biopolymer gels, including but not limited to alginate [29], fibroin [30], chitosan [31], collagen [32], and others. The introduction of CO_2_ under pressure facilitates the formation of a stable gel characterized by a homogeneous structure. Notably, one of its key advantages is the capacity to consolidate multiple stages of aerogel production within a single apparatus (gelation, solvent exchange, and supercritical drying) [29].

This investigation is devoted to examining the impact of gelation techniques on the structural attributes of aerogels. Cellulose aerogels were produced through three distinct gelation procedures coupled with supercritical carbon dioxide drying. The study also scrutinizes the influence of varying cellulose content within the initial solution on the ultimate material’s properties. An analysis of the structural characteristics of aerogels will make it possible to understand the influence of gelation methods, as well as to evaluate their potential applications. The methodology for producing cellulose aerogels using the CO_2_-induced gelation method is introduced for the first time.

## 2. Results and Discussion

**Figure 1:**
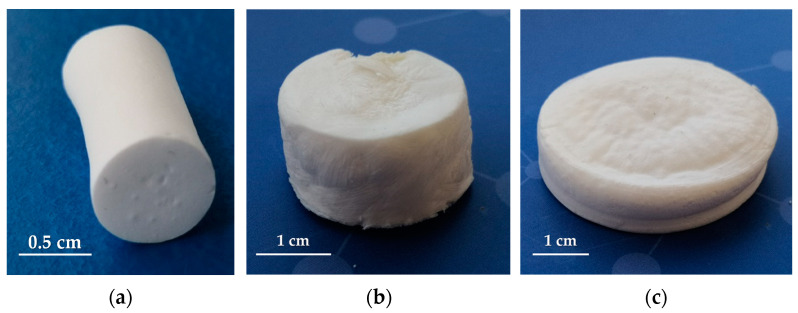
Appearance of samples obtained by: (**a**)—Chemical crosslinking; (**b**)—Cryotropic gelation; (**c**)—CO_2_-induced gelation.

This research examines the effect of the gelation technique on the structure of cellulose aerogels. Monolithic samples were fabricated through three distinct methods. The cellulose content within the initial solution spanned from 2 to 6 wt.% for each method. This range was selected given that samples tend to become fragile at lower concentrations (below 2 wt.%) and that the process becomes increasingly challenging at higher concentrations (above 6 wt.%), attributable to the high viscosity of the cellulose solution obtained in the first stage. Figure 1 shows the appearance of the obtained samples.

Table 1 presents the following structural characteristics of cellulose aerogels obtained using the three gelation methods: mass content of cellulose in the initial solution $w_{cell}$, shrinkage after the solvent exchange step $L_{gel}$, shrinkage after the supercritical drying step $L_{aero}$, bulk density of the samples $\rho_{bulk}$, skeletal density $\rho_{skelet}$, and porosity P.

**Figure 2:**
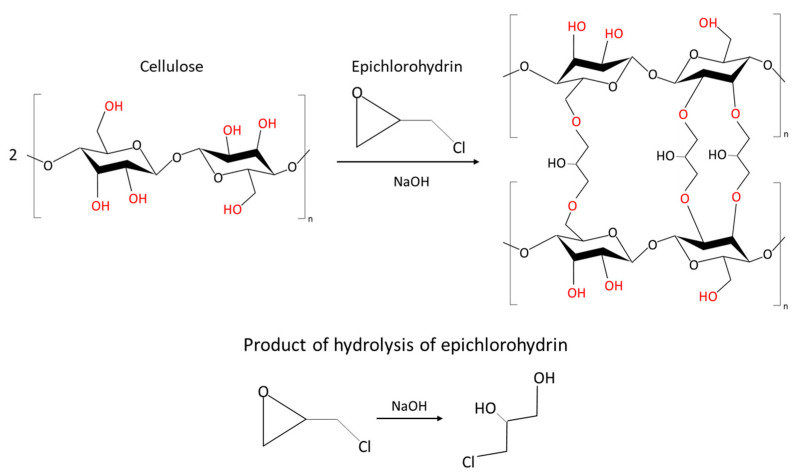
Cross-linking reaction of cellulose with epichlorohydrin in an aqueous solution under alkaline conditions.

According to the data presented in Table 1, significant shrinkage occurs during the solvent exchange stage. The strongest shrinkage and highest density are observed in samples obtained using chemical crosslinking gelation. The formation of a denser structure in this case is likely due to the presence of not only hydrogen bonds between molecules but also covalent bonds (crosslinking reaction is shown in Figure 2) [33]. The porosity of these samples is correspondingly lower than that of samples obtained without the use of crosslinking agents. An exception is sample number 1, which has the lowest shrinkage and hence the highest porosity for this method.

Porosity is one of the most important characteristics of aerogels. As $w_{cell}$ cellulose content increases, the porosity of aerogels decreases (Table 1) for all cases. However, for samples obtained without the use of crosslinkers, the porosity remains above 90% over the entire range of varying cellulose content. Thus, the methods of cryotropic gelation and CO_2_-induced gelation make it possible to obtain a highly porous material without the use of toxic compounds.

**Figure 3:**
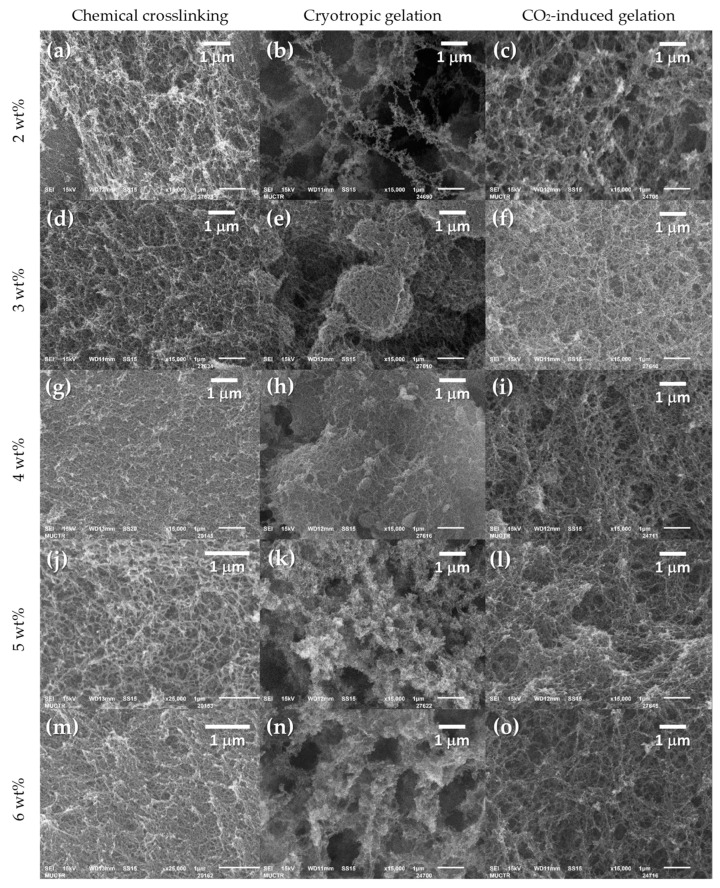
SEM images of obtained cellulose aerogels by: (**a**,**d**,**g**,**j**,**m**)—chemical crosslinking (2–6 wt.%); (**b**,**e**,**h**,**k**,**n**)—cryotropic gelation (2–6 wt.%); (**c**,**f**,**i**,**l**,**o**)—CO_2_-induced gelation (2–6 wt.%).

The scanning electron microscopy (SEM) images shown in Figure 3 demonstrate the presence of a developed mesoporous fibrous structure in the resulting aerogels.

**Figure 4:**
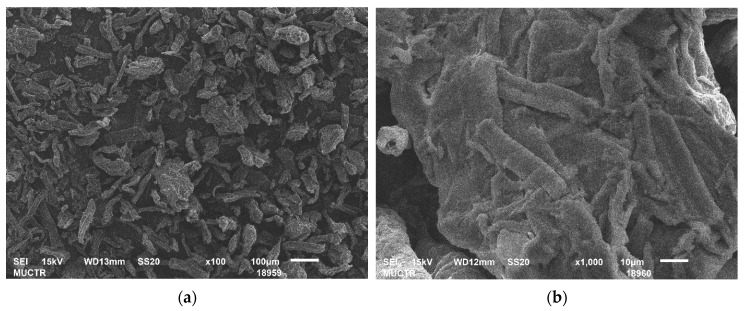
SEM-images of microcrystalline cellulose: (**a**) scale size 100 µm; (**b**) scale size 10 µm.

Figure 4 shows SEM images of microcrystalline cellulose used as a raw material. These images demonstrate the absence of a porous fibrous structure. Comparison of the SEM images in Figure 3 and Figure 4 shows that the methods used in this study promote changes in the structure of cellulose and formation of fibers.

**Figure 5:**
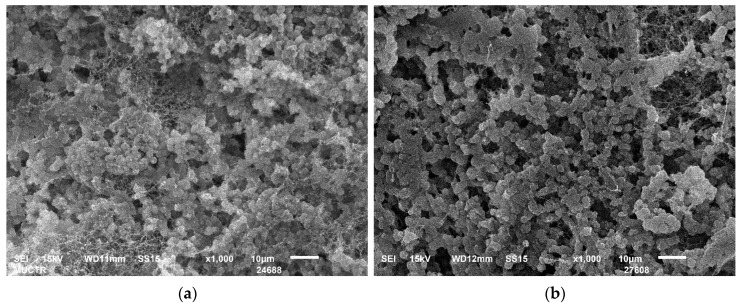
SEM images (scale size 10 µm) of aerogels obtained by cryotropic gelation: (**a**)—2 wt.%; (**b**)—3 wt.%.

In addition, samples obtained using cryotropic gelation with a low content of cellulose (2–3 wt.%) are characterized by the formation of a globular structure (Figure 5). At higher magnification (Figure 3), it can be seen that the globules are formed by fibers.

**Figure 6:**
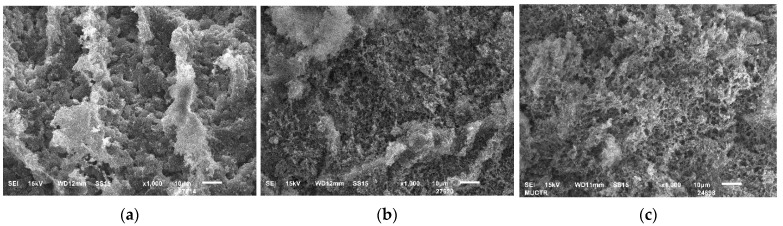
SEM images (scale size 10 µm) of aerogels obtained by cryotropic gelation: (**a**)—4 wt.%; (**b**)—5 wt.%; (**c**)—6 wt.%.

At higher cellulose content, globules are not formed (Figure 6).

**Figure 7:**
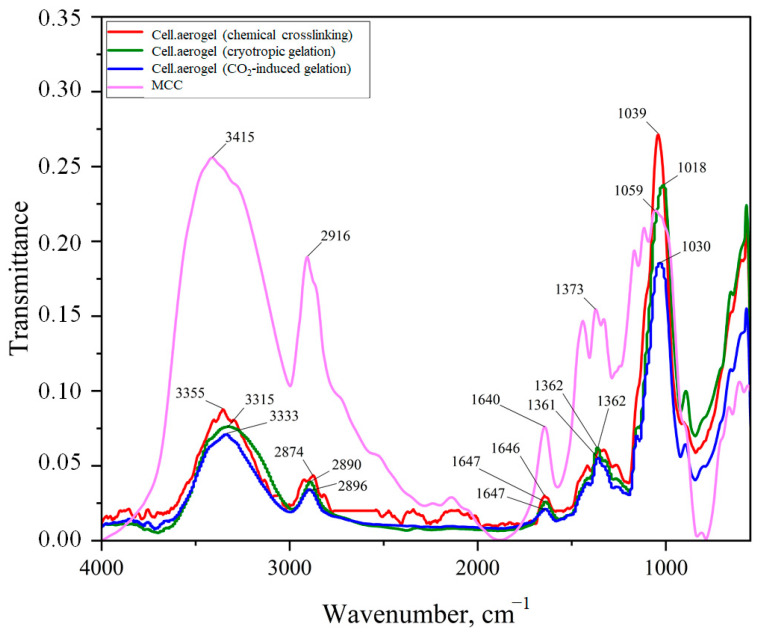
FTIR spectra of the obtained cellulose aerogels.

Figure 7 shows the Fourier-transform infrared (FTIR) spectra of the obtained cellulose aerogels and the initial microcrystalline cellulose (MCC). The observed peaks in the spectra of the aerogel samples correspond to peaks of the initial microcrystalline cellulose. However, the intensities of the obtained peaks differ. A wide band around ~3000–3600 cm^−1^, characteristic of valence vibrations of hydroxyl groups in polysaccharides, is observed in the spectra. This band also includes inter- and intramolecular hydrogen bond vibrations [34]. The intensity of this band in the aerogel samples is significantly lower than in the MCC, indicating a decrease in the number of hydrogen bonds between cellulose molecules. Peaks at wavenumbers of ∼2800–3000 cm^−1^ correspond to the vibrations of C–H bonds in all carbohydrate components, and peaks at ∼1400–1300 cm^−1^ correspond to the OH–CH bond. Their intensity in the aerogels is lower than in the MCC. Vibrations of C–O–C are observed at ~1310–1000 cm^−1^ [35]. The aerogel obtained using chemical crosslinking has the highest intensity in this position. This is due to the formation of new bonds between cellulose molecules, as shown in Figure 2. In addition, all samples have a peak at ~1650–1630 cm^−1^, corresponding to the vibrations of crystallization water, i.e., the intensity is related to the moisture content in the samples. The moisture content in the aerogel samples is lower than in the initial MCC.

**Figure 8:**
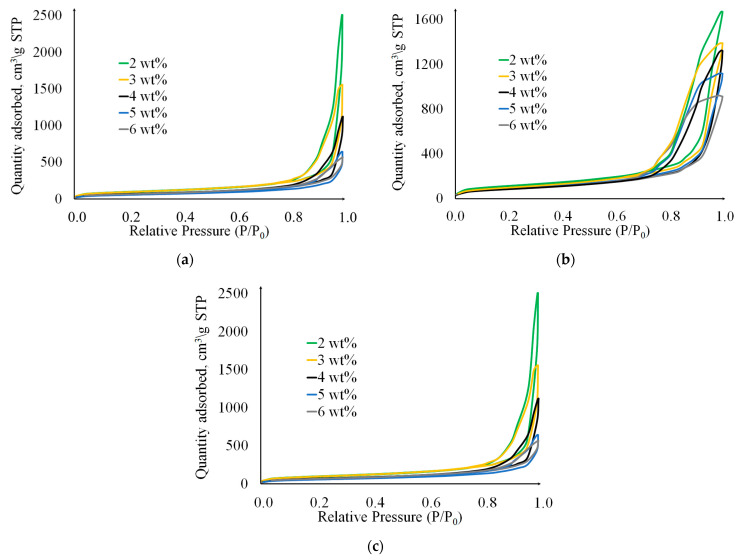
Nitrogen adsorption/desorption isotherms at 77 K for cellulose aerogels prepared using the methods: (**a**)—Chemical crosslinking; (**b**)—Cryotropic gelation; (**c**)—CO_2_-induced gelation.

Analysis of the porous structure of cellulose aerogels was carried out using physical nitrogen sorption. Nitrogen adsorption-desorption isotherms at 77 K are presented in Figure 8. According to the International Union of Pure and Applied Chemistry (IUPAC) classification [36], the obtained isotherms belong to Type IV. This type is typical for mesoporous materials, and the presence of hysteresis loops indicates the occurrence of capillary condensation. For samples obtained using chemical cross-linking, the amount of adsorbed nitrogen is lower compared with the other methods, indicating a lower total pore volume. For samples obtained by cryotropic gelation and pressure gelation methods, the amount of adsorbed nitrogen decreases with increasing cellulose content.

**Figure 9:**
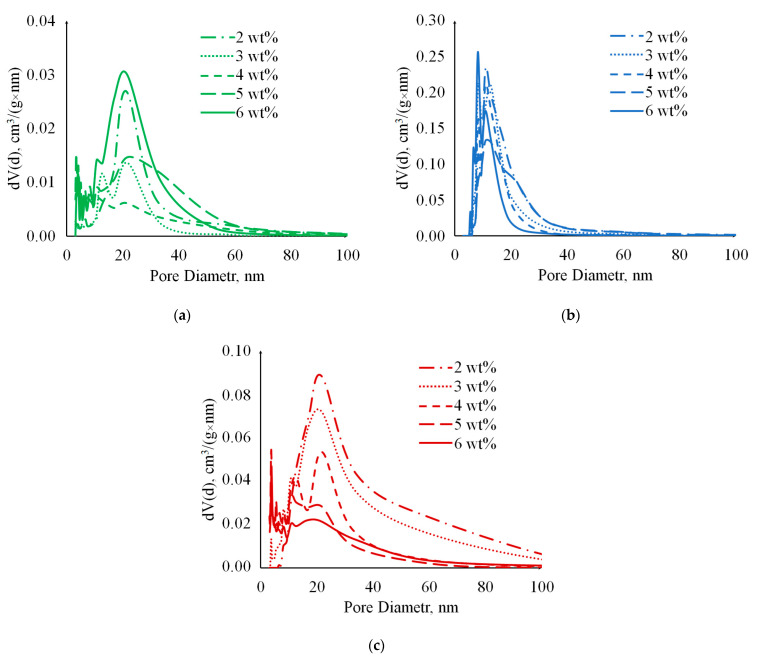
Pore size distribution of cellulose aerogels prepared using the methods: (**a**)—Chemical crosslinking; (**b**)—Cryotropic gelation; (**c**)—CO_2_-induced gelation.

Figure 9 shows the differential pore size distribution curves obtained by the Barrett-Joyner-Halenda (BJH) method, demonstrating the predominance of the mesoporous structure.

For samples obtained using cryotropic gelation, a narrower pore distribution is characteristic (Figure 9b) compared with the other methods. For samples obtained by pressure gelation, an increase in cellulose content is associated with a decrease in pore volume and a shift of peaks towards pores with smaller diameters.

Table 2 shows the specific surface area $S_{BET}$, obtained by the Brunauer-Emmett-Teller (BET) method, and the mesopore volume $V_{BJH}$, obtained by BJH method.

The values of $S_{BET}$ and $V_{BJH}$ for samples obtained using chemical cross-linking, as shown in Table 2, are significantly lower than those obtained by other methods. It should be noted that methods without the use of cross-linking agents allow for obtaining materials with a controlled structure. As seen from Table 2, with an increase in cellulose content, the parameters $S_{BET}$ and $V_{BJH}$ decrease. However, for samples obtained by chemical cross-linking, no clear dependence is observed.

## 3. Conclusions

Cellulose is of great interest for obtaining aerogels due to its environmentally friendly and renewable attributes. The application of diverse techniques enables the creation of materials with tailored properties. This study scrutinized the impact of gel formation methods on the structural attributes of cellulose aerogels. Three distinct gel formation methods were employed: chemical cross-linking, cryotropic gelation, and CO_2_-induced gelation. The developed approaches demonstrated the ability to yield stable cellulose gels. The resultant samples exhibited a mesoporous and fibrous structure. Notably, chemical cross-linking led to the formation of a denser structure (0.067–0.349 g/cm^3^), consequently reducing porosity (77.2–95.8%) and specific surface area (33–87 m^2^/g). In contrast, cryotropic gelation and pressure-induced gelation facilitated the production of aerogels with higher porosity (>91%) and specific surface area (225–406 m^2^/g) without the necessity of a cross-linking agent. The absence of a cross-linking agent is particularly pertinent for applications requiring material biocompatibility, as these agents often involve the use of toxic substances. This study underscores the capacity of gel formation methods to influence the characteristics of resulting aerogels.

## 4. Materials and Methods

### 4.1. Materials

MCC was used as a source of cellulose. Granulated sodium hydroxide (RusHim, Russia), urea (RusHim, Moscow, Russia), and distilled water obtained in the laboratory were used to obtain the cellulose solvent. Epichlorohydrin (ECH, Sigma-Aldrich, St. Louis, MO, USA) was used as a cross-linking agent. Ethanol (RusHim, Russia) was used for cellulose regeneration. Isopropyl alcohol (IPA, RusHim, Russia) was used as the solvent.

### 4.2. Methods

#### 4.2.1. Cellulose Dissolution

The first step in obtaining cellulose-based aerogels is the dissolution of MCC. A 7 wt.% NaOH/12 wt.% (NH_2_)_2_CO aqueous solution was used as the cellulose solvent. To prepare it, sodium hydroxide and urea were dissolved in distilled water, then cooled in a freezer (−12 °C) for about 1 h. The MCC was added to the solution, and dispersion was carried out at 10,000 rpm for 5 min using an IKA T 25 digital ULTRA-TURRAX homogenizer (IKA-Werke, Staufen, Germany).

#### 4.2.2. Preparation of Cellulose Hydrogels

**Figure 10:**
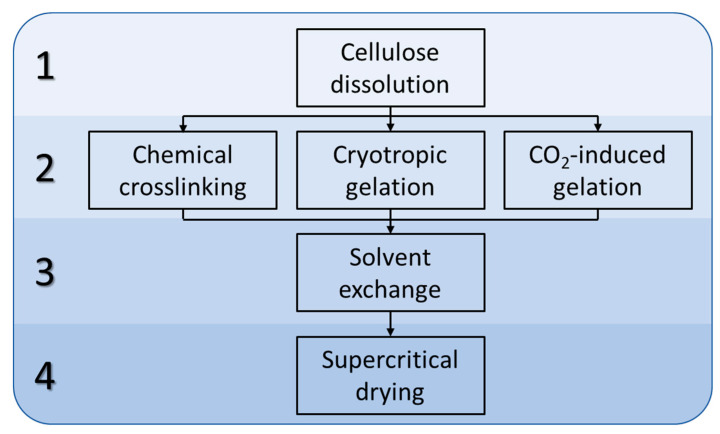
General scheme for the production of cellulose aerogels.

The following approaches were used to prepare cellulose gels: chemical cross-linking, cryotropic gelation, and pressure gelation (Figure 10).

##### Chemical Cross-Linking Gelation

The obtained cellulose solution was mixed with the cross-linking agent epichlorohydrin, and the solution was homogenized at 6000 rpm for 8 min before being poured into cylindrical molds with a volume of 3 mL and an inner diameter of 12.5 mm. The samples were left at room temperature for one day until the gelation process was complete.

##### Cryotropic Gelation

The obtained cellulose solution was poured into Petri dishes, which were placed in a freezer at −26 °C for 24 h. The resulting monoliths were immersed in ethanol to facilitate the gelation process.

##### CO_2_-Induced Gelation

The obtained cellulose solution was poured into Petri dishes, which were placed in a 2-L high-pressure vessel. The gelation process was carried out in a CO_2_ environment at a pressure of 50 bar and at room temperature. The process was conducted under constant pressure for 24 h. Pressure release was carried out at a rate of 1–2 bar/min to avoid bubble formation inside the gel.

#### 4.2.3. Solvent Exchange

After the gelation process, it is important to remove all unreacted reagents and ensure complete solvent exchange in the pores. To remove unreacted reagents, the samples were first immersed in distilled water. Water exchange was performed until reaching a neutral pH value.

Water is not soluble in supercritical carbon dioxide. To facilitate the subsequent supercritical drying process, the water in the gel pores was replaced with IPA. To avoid excessive shrinkage of the samples, a stepwise solvent exchange was performed with gradual increase in IPA concentration (20% → 40% → 60% → 80% → 100%). The intervals between the exchange steps were at least 4 h. The final exchange step (100%) was performed twice to ensure complete replacement of the solvent in the gel pores.

#### 4.2.4. Drying

Supercritical drying of the samples was carried out in a self-designed high-pressure reactor. The process conditions were a temperature of 40 °C and pressure of 120–140 bar. The drying time was 4–6 h.

**Figure 11:**
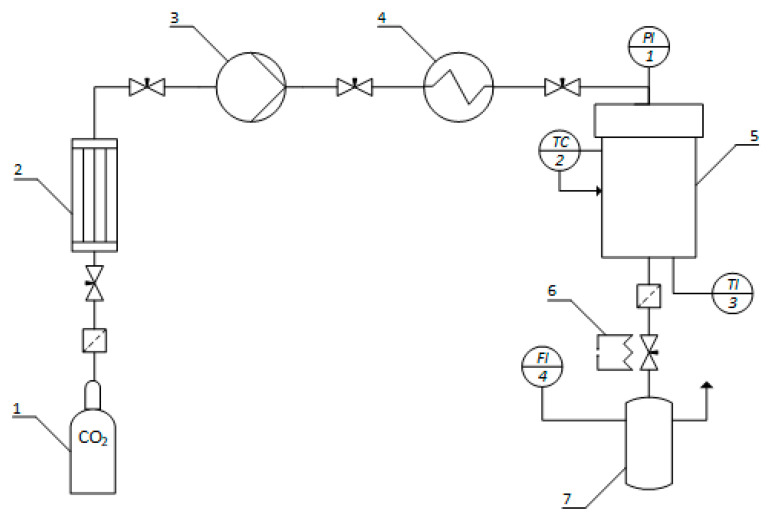
The diagram of the installation for CO_2_-induced gelation and supercritical drying is as follows: 1—carbon dioxide cylinder (60 atm); 2—condenser; 3—pump; 4—heat exchanger; 5—2-L high-pressure vessel; 6—heating element; 7—separator.

The technological scheme of the installation for pressure-induced gelation and supercritical drying is shown in Figure 11. Carbon dioxide, in liquid state at 60 atm, is supplied from cylinder 1 to condenser 2 for cooling and prevention of gasification. In the high-pressure vessel 5, preheated in heat exchanger 4, carbon dioxide is delivered by pump 3 at a certain pressure. Inside the apparatus, temperature sensor TI3 and pressure gauge PI1 are installed. Temperature regulation is carried out by thermostat TC2. The flow rate of the outgoing stream is controlled by a system of valves at the outlet of the apparatus. A flowmeter FI4 is used to measure the flow rate of gaseous carbon dioxide.

#### 4.2.5. Characterization

The obtained samples were analyzed using various methods to determine their structural characteristics. The true density of the samples was determined by helium pycnometry using the Anton Paar UltraPyc 5000 (Anton Paar GmbH, Austria) instrument. The porosity P of the obtained samples was determined using Formula (1):

(1) $$P=\left(1-\frac{\rho_{bulk}}{\rho_{skelet}}\right)\cdot 100\%,$$

where $\rho_{bulk}$ is the apparent density, g/cm^3^; $\rho_{skelet}$ is the true density of the cellulose fiber, g/cm^3^.

The porous structure of the samples was investigated using low-temperature nitrogen adsorption (77 K) on the NOVA 2200E specific surface area analyzer (Quantachrome Instruments Corp., Boynton Beach, FL, USA). Before analysis, the samples were pre-treated at a pressure of 0.5 mmHg and a temperature of 353 K for 12 h to remove all adsorbed moisture from the surface. The specific surface area was determined by the BET method, and the pore size distribution and mesopore volume were determined by the BJH method.

The infrared absorption spectrum was obtained using the Nicolet380 Fourier Transform Infrared Spectrometer (Thermo Fisher Scientific Inc., Waltham, MA, USA) at the Shared Facility Center of Mendeleev University. The spectra of cellulose-based aerogel samples were recorded in the range of 4000 to 500 cm^−1^.

The morphology of the material was investigated using SEM on the JEOL 1610LV equipment (JEOL, Tokyo, Japan) at the Mendeleev University Shared Facility Center.

The linear shrinkage of the samples was determined based on their linear dimensions before and after supercritical drying. To evaluate it, the diameter of the samples was measured using calipers. The linear shrinkage L was determined by Formula (2):

(2) $$L=\frac{d_{i-1}-d_{i}\;}{d_{i-1}}\cdot 100\%,$$

where $d_{i}$ is the diameter of the sample at the i-th stage; $d_{i-1}$ is the diameter of the sample at the previous i-th stage.

## Figures and Tables

**Table 1:** Structural Characteristics of Cellulose Aerogels.

№	Gelation Method	$w_{cell}$, wt%	$L_{gel}$, %	$L_{aero}$, %	$\rho_{bulk}$, g/cm^3^	$\rho_{skelet}$, g/cm^3^	P, %
1	Chemical crosslinking	2.0	21 ± 3.0	16 ± 1.6	0.067 ± 0.004	1.589 ± 0.021	95.8
2		3.0	33 ± 3.6	8 ± 1.4	0.189 ± 0.007	1.488 ± 0.013	87.3
3		4.0	31 ± 1.1	11 ± 0.9	0.201 ± 0.011	1.592 ± 0.017	87.4
4		5.0	30 ± 1.0	7 ± 0.7	0.214 ± 0.013	1.632 ± 0.007	86.9
5		6.0	31 ± 0.9	11 ± 0.8	0.349 ± 0.017	1.530 ± 0.009	77.2
6	Cryotropic gelation	2.0	24 ± 0.4	9 ± 1.0	0.047 ± 0.007	1.856 ± 0.004	97.5
7		3.0	24 ± 0.7	11 ± 0.8	0.084 ± 0.005	1.870 ± 0.007	95.5
8		4.0	25 ± 0.4	5 ± 0.8	0.094 ± 0.003	1.648 ± 0.011	94.3
9		5.0	24 ± 0.3	7 ± 1.3	0.126 ± 0.005	1.737 ± 0.014	92.8
10		6.0	22 ± 0.4	2 ± 0.9	0.131 ± 0.001	1.565 ± 0.008	91.6
11	CO_2_-induced gelation	2.0	16 ± 0.5	9 ± 0.5	0.045 ± 0.002	1.634 ± 0.010	97.2
12		3.0	17 ± 0.9	4 ± 0.9	0.059 ± 0.000	2.176 ± 0.005	97.3
13		4.0	17 ± 0.4	9 ± 0.4	0.073 ± 0.000	1.603 ± 0.016	95.5
14		5.0	17 ± 0.2	5 ± 0.5	0.101 ± 0.000	1.961 ± 0.004	94.9
15		6.0	15 ± 0.2	7 ± 0.6	0.108 ± 0.000	1.606 ± 0.013	93.3

**Table 2:** Nitrogen porosimetry results.

№	Gelation Method	$w_{cell}$, wt%	$S_{BET}$, м^2^/г	$V_{BJH}$, cм^3^/г
1	Chemical crosslinking	2.0	63 ± 7	0.34 ± 0.06
2		3.0	33 ± 7	0.23 ± 0.03
3		4.0	58 ± 11	0.18 ± 0.07
4		5.0	87 ± 46	0.31 ± 0.21
5		6.0	78 ± 34	0.36 ± 0.23
6	Cryotropic gelation	2.0	406 ± 8	2.64 ± 0.12
7		3.0	379 ± 7	2.21 ± 0.16
8		4.0	332 ± 5	2.08 ± 0.11
9		5.0	326 ± 5	1.95 ± 0.15
10		6.0	312 ± 8	1.54 ± 0.10
11	CO_2_-induced gelation	2.0	343 ± 11	3.70 ± 0.26
12		3.0	325 ± 21	2.19 ± 0.24
13		4.0	281 ± 14	1.88 ± 0.13
14		5.0	232 ± 19	1.13 ± 0.14
15		6.0	225 ± 13	0.82 ± 0.04

## Data Availability

The data presented in this study are openly available in article.
